# Recent Insights into the Role of Hypothalamic AMPK Signaling Cascade upon Metabolic Control

**DOI:** 10.3389/fnins.2012.00185

**Published:** 2012-12-20

**Authors:** Marc Schneeberger, Marc Claret

**Affiliations:** ^1^Diabetes and Obesity Laboratory, Institut d’Investigacions Biomèdiques August Pi i SunyerBarcelona, Spain; ^2^Centro de Investigación Biomédica en Red de Diabetes y Enfermedades Metabólicas AsociadasBarcelona, Spain; ^3^Department of Endocrinology and Nutrition, Hospital Clínic, School of Medicine, University of BarcelonaBarcelona, Spain

**Keywords:** AMPK, hypothalamus, appetite, thermogenesis, obesity, glucose metabolism, energy balance

## Abstract

In 2004, two seminal papers focused on the role of AMP-activated protein kinase (AMPK) in the hypothalamus opened new avenues of research in the field of the central regulation of energy homeostasis. Over the following 8 years, hundreds of studies have firmly established hypothalamic AMPK as a key sensor and integrator of hormonal and nutritional signals with neurochemical and neurophysiological responses to regulate whole-body energy balance. In this review article we aim to discuss the most recent findings in this particular area of research, highlighting the function of hypothalamic AMPK in appetite, thermogenesis, and peripheral glucose metabolism. The diversity of mechanisms by which hypothalamic AMPK regulates energy homeostasis illustrates the importance of this evolutionary-conserved energy signaling cascade in the control of this complex and fundamental biological process.

## Introduction

AMP-activated protein kinase is an evolutionary-conserved heterotrimeric serine/threonine protein kinase that acts as a cellular and organismal energy sensor, regulating systemic metabolism through multiple effects in different tissues (Hardie et al., 2012). In the hypothalamus, AMPK is a central mediator of the adaptive responses to physiological regulation of feeding behavior through the integration of hormonal and nutritional cues (Blanco Martinez de Morentin et al., 2011). In general terms, orexigenic signals (fasting, hypoglycemia, ghrelin, etc.) in the hypothalamus activate AMPK, while anorexigenic signals (feeding, hyperglycemia, insulin, leptin, etc.) exert the opposite effect (Minokoshi et al., 2004; Blanco Martinez de Morentin et al., 2011). However, the hypothalamus is composed of different nuclei and neuronal populations with specific functions, and therefore it is important to delineate the role of AMPK in discrete subpopulations of neurons. In an attempt to address this, we generated mice with a conditional deletion of the α2 catalytic subunit of AMPK in key neuronal populations of the arcuate nucleus of the hypothalamus (ARC), namely pro-opiomelanocortin (POMC) and agouti-related protein (AgRP) neurons (Claret et al., 2007). Our data demonstrated critical but divergent effects on energy balance when AMPK was deleted in POMC or AgRP neurons, suggesting that AMPK plays specific roles in particular populations of neurons rather than being a general integrator of energy homeostasis in the hypothalamus. Furthermore, our study also revealed an essential glucose-sensing function for AMPK in both POMC and AgRP neurons (Claret et al., 2007). Although studies targeting discrete populations of neurons are further required, the bulk of studies performed to date firmly establish hypothalamic AMPK as a critical sensor and integrator of nutrient and hormonal-related signals implicated in whole-body energy homeostasis. The aim of this review article is not to exhaustively discuss the role of hypothalamic AMPK in energy homeostasis control, but to introduce the most recent findings and concepts exploring this fundamental regulatory system.

## Hypothalamic AMPK and Appetite Regulation

**Figure 1:**
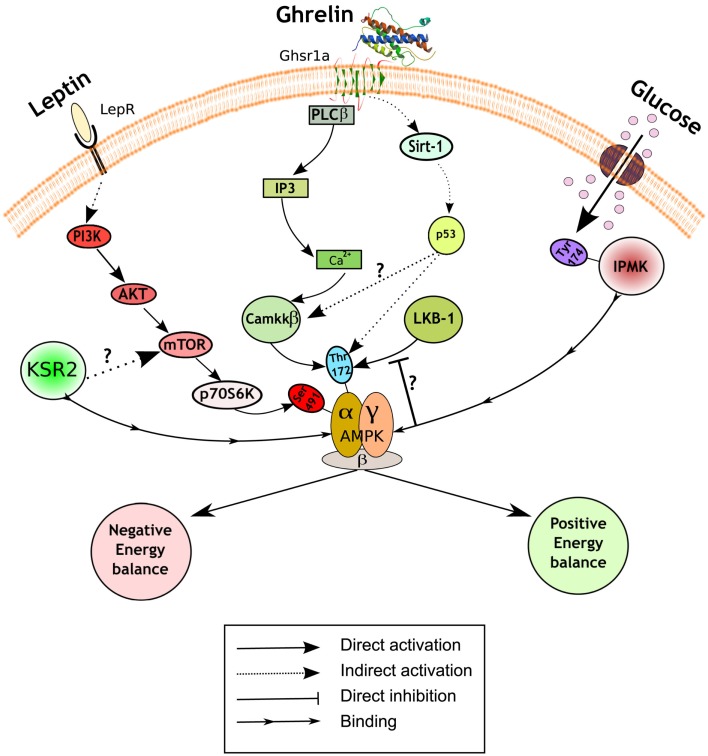
**Hypothalamic AMPK regulates appetite**. Schematic representation of a generic hypothalamic neuron depicting a summary of the recently uncovered signaling mechanisms implicated in appetite control. Pathways in red inhibit AMPK activity and subsequently reduce food intake and body weight (negative energy balance), while pathways in green activate it and lead to the opposite physiological outputs (positive energy balance). A diagram of the role of presynaptic AMPK can be found elsewhere (Yang et al., 2011; Hardie et al., 2012). LepR, leptin receptor; Ghsr1a, growth hormone secretagogue receptor type 1a; PI3K, phosphatidylinositol 3-kinase; Akt, protein kinase B; mTOR, mammalian target of rapamycin; KSR2, kinase suppressor of Ras 2; PLCβ, phospholipase C; IP3, inositol-1,4,5-triphosphate; Ca^2+^, calcium; LKB1, liver kinase B1; CamKKβ, Ca^2+^/Calmodulin kinase kinase β; Sirt-1, sirtuin 1; IPMK, inositol polyphosphate multikinase.

The regulation of AMPK activity is complex, but mainly achieved through phosphorylation of threonine^172^ on the catalytic α subunit by upstream kinases, such as liver kinase B1 (LKB1) and Ca^2+^/Calmodulin kinase kinase β (CamKKβ; Hardie et al., 2012). A recent study has reported that inositol polyphosphate multikinase (IPMK) may be an upstream physiologic regulator of AMPK activity in the hypothalamus (Bang et al., 2012). IPMK inhibits AMPK activity during the fasting/refed transition through binding between these two proteins, an interaction facilitated by IPMK phosphorylation on tyrosine^174^. The working hypothesis is that the AMPK/IPMK complex is a worse substrate than AMPK alone for upstream kinases such as LKB1, and/or is more susceptible to protein phosphatases thus preventing AMPK phosphorylation and activation (Figure 1). The binding of IPMK to AMPK might represent an additional and perhaps physiologically relevant mechanism of AMPK activity regulation. In this regard, it would be interesting to explore the identity of the kinase that phosphorylates IPMK, and also if this event is also modulated by metabolic hormones or other nutrients. These findings emphasize the importance of a fine-tuning balance of the phosphorylation status of threonine^172^ to exert the diversity of AMPK functions, and suggest a potential target to modulate AMPK activity.

Another putative regulator of AMPK activity is kinase suppressor of Ras 2 (KSR2). This protein likely functions as a molecular scaffolding, rather than a kinase, that binds to AMPK (Costanzo-Garvey et al., 2009; Liu et al., 2009). Two studies have reported the metabolic consequences of global KSR2 deletion with discordant results. Costanzo-Garvey et al. (2009) suggest that the observed obesity phenotype in KSR2 knock-out mice is due to reduced fatty acid oxidation and thermogenesis. In contrast, Revelli et al. (2011) present evidence of increased body weight mainly due to profound hyperphagia. Furthermore, while the former report links the phenotype to alterations in AMPK activity, the latter study suggests the involvement of mTOR in this process (Figure 1). These differences in phenotype may be the result of different genetic backgrounds and/or gene-targeting events. Additional studies will be required to assess the relevance of KSR2 as a regulator of AMPK signaling cascade and nutrient sensor in the hypothalamus.

Some of the most exciting recent findings on the role of hypothalamic AMPK upon energy balance control are related with the orexigenic hormone ghrelin. The central effects of ghrelin on food intake and body weight are mediated by AMPK activation in AgRP neurons of the ARC (Andrews et al., 2008; Lopez et al., 2008). The current model of action propounds that ghrelin, after binding its receptor (GHSR1a), increases intracellular Ca^2+^ stimulating AMPK activity via CamKKβ activation. However, our knowledge of the precise molecular mechanisms implicated is still incomplete. Velasquez et al. have recently reported a novel sequence of events between the ghrelin receptor and AMPK activation that further describes this pathway. Using a combination of pharmacological and genetic approaches, the authors demonstrate that the orexigenic action of ghrelin is mediated by a sirtuin 1 (SIRT-1)-p53-AMPK pathway (Velasquez et al., 2011; Figure 1). It is unknown whether this pathway converges somehow with CamKKβ or if it is part of a parallel signaling pathway mediating slightly different physiological ghrelin effects. Furthermore, this study challenges the field suggesting a potential role for p53, a tumor-suppressor protein widely implicated in cancer, in the central regulation of metabolism. The study of conditional knock-outs of p53 in specific populations of neurons will help to expand these exciting findings.

In contrast to the aforementioned prevailing view, supporting that ghrelin directly acts on AgRP neurons to exert its orexigenic actions, a recent report provides an additional layer of complexity to the current model. Yang et al. (2011) have used an elegant combination of electrophysiology, pharmacology, and optogenetics to unveil a novel presynaptic pathway that stimulates the activity of AgRP neurons. The authors provide evidence indicating that, under fasting conditions, ghrelin increases presynaptic AMPK activity through intracellular Ca^2+^ release and subsequent CamKKβ activation. Elevated AMPK activity is able to further release Ca^2+^ via ryanodine receptors, thus causing a positive feedback loop and synaptic hysteresis onto AgRP neurons. This sustained synaptic upregulation induced by ghrelin can be reversed by the anorexigenic adipokine leptin, which activates POMC neurons and resets the system by releasing a still undefined opioid. These results highlight the relevance of AMPK in presynaptic neurons, but do not formally exclude the existence of a key and complementary role for postsynaptic AMPK in the regulation of energy balance *in vivo*. In fact, previous studies have shown a key cell-autonomous role for AMPK in both AgRP and POMC neurons (Claret et al., 2007). Furthermore, hysteresis for AMPK does not occur in all cells, so it is likely that appropriate regulation of appetite and body weight is achieved by a combination of presynaptic and postsynaptic events.

The adipokine leptin is another crucial hormone implicated in the central regulation of energy balance that exerts its actions through AMPK. Leptin inhibits AMPK activity in the hypothalamus (Andersson et al., 2004; Minokoshi et al., 2004), an effect that is in contrast with its role in the periphery (Minokoshi et al., 2002). The signaling events implicated in leptin-induced AMPK inhibition in the hypothalamus have been recently uncovered by Dagon et al. (2012). The authors found that phosphorylation on serine^485/491^ of AMPKα2 was associated with reduced AMPK activity in the ARC, ventromedial hypothalamus (VMH), and paraventricular nucleus (PVN). This AMPK inhibition, which was sufficient to regulate appetite and body weight, was independent of the phosphorylation status of threonine^172^. These results suggest that serine^485/491^ may act as an “off switch,” adding a further level of regulatory sophistication to this already complex system. Interestingly, p70S6K is the kinase responsible for this inhibitory phosphorylation on AMPK. Together, these results suggest the existence of a coordinated leptin-stimulated signaling network in the hypothalamus that unifies phosphatidylinositol 3-kinase (PI3K)-AKT, mammalian target of rapamycin (mTOR)-p70S6K, and AMPK.

## Hypothalamic AMPK and Regulation of Glucose Metabolism

**Figure 2:**
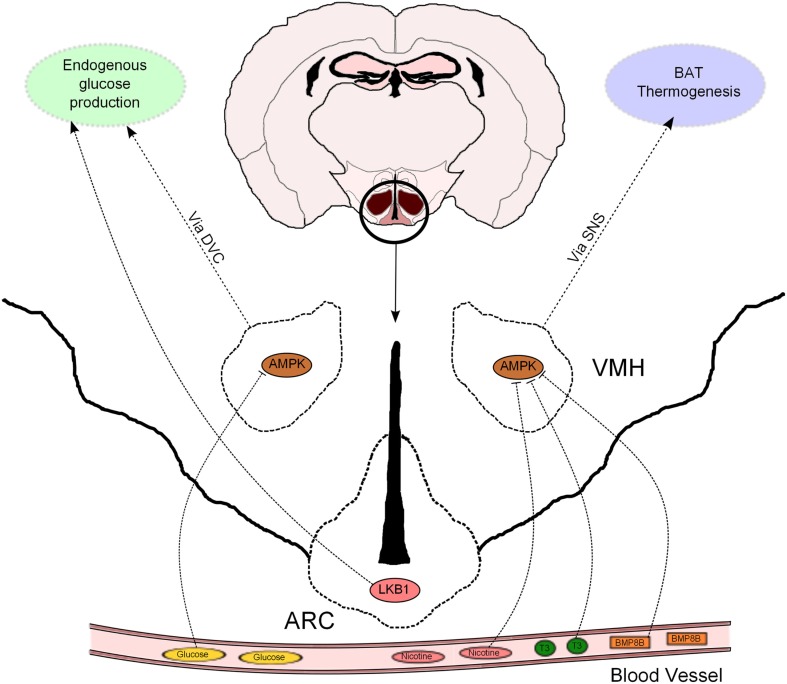
**Hypothalamic AMPK regulates glucose metabolism and thermogenesis**. Nicotine, triiodothyronine (T3), and bone morphogenetic protein 8B (BMP8B) exert their thermogenic properties on brown adipose tissue (BAT) through inhibition of AMPK in the ventromedial hypothalamus (VMH). AMPK in the VMH also acts as a glucose sensor to modulate endogenous glucose production. This effect is mediated, at least partially, by the dorsal vagal complex (DVC). LKB1 in arcuate (ARC) POMC neurons regulate systemic glucose metabolism through hepatic glucose production modulation.

The hypothalamus has emerged as a relevant determinant of whole-body glucose homeostasis through different effects in muscle, pancreas, and liver (Marino et al., 2011). In particular, the role of hormonal signaling and nutrient sensing in the hypothalamic control of endogenous glucose production (EGP) has recently received increasing attention. In this regard, AMPK has been proposed to mediate some of these effects. For example, AMPK in the VMH has been implicated in hypoglycemia sensing and also in non-hormonal, as well as hormonal, counterregulatory responses to stimulate glucose production (McCrimmon et al., 2004, 2006, 2008). A novel study, using genetic tools allowing bidirectional changes in AMPK activity, confirms, and extends these findings (Yang et al., 2010; Figure 2). Mediobasal hypothalamic injection of a dominant negative (DN) isoform of AMPKα was sufficient to suppress glucose production in rats. Although the delivery of a constitutive active (CA) AMPKα form did not produce the opposite effect, it was able to negate the hypothalamic glucose/lactate-sensing mechanisms to reduce glucose production (Yang et al., 2010). These findings, which were independent of changes in the concentration of circulating pancreatic hormones, suggest that hypothalamic AMPK is sufficient and necessary to regulate EGP. However, it is important to bear in mind that this study was conducted using the pancreatic euglycemic clamp technique, so the relevance of this regulatory system should be further assessed in a more physiological setting.

It has been recently proposed that the effects of hypothalamic AMPK on EGP are downstream mediated by neurons located at the dorsal vagal complex (DVC) within the hindbrain. *N*-methyl-d-aspartate (NMDA) receptor has been implicated in the intestine nutrient sensing mechanisms regulating glucose production (Lam et al., 2010). Interestingly, pharmacological and genetic inhibition of NMDA receptor in the DVC blunted the ability of a hypothalamic DN AMPK form to reduce glucose production (Lam et al., 2011). This study strengthens the notion that DVC NMDA receptor-expressing neurons integrate information from nutrient signals in order to regulate glucose homeostasis via AMPK, but does not exclude the implication of other neuronal systems in this biological process. In fact, the hypothalamus can influence additional autonomic nuclei to control whole-body metabolism (Marino et al., 2011), and thus further investigation is needed to uncover the extrahypothalamic neuronal networks that may be involved in the hypothalamic AMPK control of glucose production.

Leptin and insulin signaling in ARC POMC and AgRP neurons also regulates hepatic glucose fluxes (Marino et al., 2011). We have previously shown that POMC and AgRP neurons lacking AMPKα2 became insensitive to alterations in extracellular glucose concentrations, a defect that was associated with alterations in whole-body energy balance but not glucose metabolism (Claret et al., 2007). In this regard, recent evidence further indicates that AMPKα2 is a sensor of glucose fluctuations in a hypothalamic cell line, possibly through the modulation of uncoupling protein 2 (UCP2) activity (Beall et al., 2012). Interestingly, POMC neurons lacking the upstream kinase LKB1 exhibited normal glucose-sensing, but these mice showed systemic glucose intolerance, insulin resistance and impaired HGP with concomitant upregulation of key gluconeogenic enzymes (Claret et al., 2011; Figure 2). These metabolic defects were associated with reduced alpha-melanocyte stimulating hormone (α-MSH) secretion from POMC neurons. Remarkably, deletion of CamKKβ in POMC neurons did not produce any obvious metabolic phenotype (Claret et al., 2011).

These divergences in phenotypes can be explained by the fact that LKB1 is also able to signal through several AMPK-related kinases other than AMPK. In addition, compensatory mechanisms aimed to maintain the functionality of the signaling cascade could also explain these disparities. Similar *in vivo* examples of phenotype divergence can be found in studies targeting AMPK (Beall et al., 2010; Sun et al., 2010a) or LKB1 (Fu et al., 2009; Granot et al., 2009; Sun et al., 2010b) in pancreatic β-cells. Taken together our data support the idea that, at least in POMC neurons, AMPK might be implicated in feeding and energy expenditure regulation whereas LKB1 would regulate alternative downstream effectors to control glucose metabolism. This data also suggest that systemic glucose homeostasis does not necessarily correlate with glucose-sensing in POMC neurons.

## Hypothalamic AMPK and Regulation of Thermogenesis

Thermogenesis in brown adipose tissue (BAT) is an essential component of the energy balance equation. It has been recognized for a long time that the hypothalamus, and in particular the VMH, plays a crucial role in the regulation of BAT activity (Cannon and Nedergaard, 2004). However, the molecular mechanisms mediating these effects have been elusive for a long time. A number of excellent reports from the same research group have recently proposed hypothalamic AMPK as a pivotal thermogenic regulator of BAT through the sympathetic nervous system (SNS).

Whittle and collaborators have studied the metabolic role of bone morphogenetic protein 8B (BMP8B). This protein was highly expressed in brown adipocytes and its expression modulated by different thermogenic stimuli (Whittle et al., 2012). Mice globally lacking BMP8B exhibited reduced thermogenesis, increased body weight, and enhanced propensity for weight gain when fed with high-fat diet (HFD). BMP8B was also found to be expressed in the brain and particularly enriched in the ARC and VMH. Interestingly, the authors observed attenuated activation of AMPKα in the hypothalamus from knock-out mice under basal conditions. Intracerebroventricular (ICV) administration of BMP8B activated VMH neurons and BAT thermogenesis in rodents. Targeted delivery of a DN isoform of AMPKα in the VMH resulted in a higher thermogenic effect of ICV BMP8B administration, whereas expression of CA AMPKα isoform blunted BMP8B-stimulated activation of BAT (Whittle et al., 2012). These results demonstrate that the central thermogenic action of BMP8B is dependant on hypothalamic AMPK activity (Figure 2). It is important to note that changes in VMH AMPK activity only produced thermogenic effects in combination with BMP8B injection, suggesting that these two proteins may act as a counterregulatory mechanism to modulate BAT thermogenesis.

Hypothalamic AMPK has also been critically implicated in the thermogenic response elicited by thyroid hormones. Lopez et al. (2010) provided compelling evidence that hyperthyroidism and ICV triiodothyronine (T3) injection inactivate hypothalamic AMPK, stimulating *de novo* lipogenesis, and BAT activity through the SNS (Figure 2). These effects were inhibited when a CA AMPKα isoform was specifically delivered to the VMH, indicating that inhibition of VMH AMPK by thyroid hormones is necessary to perform their metabolic effects. Together, these evidences highlight the pathophysiological relevance of this central mechanism in hyperthyroidism and provide important conceptual advances in the understanding of this pathology.

The AMPK-BAT axis has also been proposed to mediate the anorexigenic properties of nicotine. These effects, in both humans and rodents, have been mostly attributed to central actions but the molecular mechanisms implicated are poorly understood. Martinez de Morentin et al. (2012) demonstrate that the negative energy balance (reduced food intake and increased BAT activity) induced by nicotine treatment was associated with hypothalamic AMPK inhibition and changes in neuropeptide expression. A combination of pharmacological and genetic approaches proved, similarly to previous studies, that AMPK in the VMH played a key role in mediating the nicotine effects, not only on BAT thermogenesis but also on food intake. In this sense, it is interesting to note that, depending on the modulatory affector, AMPK in the VMH is able to either target BAT thermogenesis (as in hyperthyroidism) or both food intake and energy expenditure (after nicotine treatment). Therefore, it remains to be elucidated the differential modulatory effects on AMPK activity and/or signaling pathways downstream of AMPK that are able to dissociate these effects.

Collectively, these studies show that different thermogenic stimuli mediate their effects through modulation of AMPK activity in the VMH. Thus, it is tempting to speculate that the VMH AMPK-BAT axis may be a general regulator of BAT activity implicated in energy homeostasis control. Given the current evidence highlighting the importance of BAT in adult humans (Ravussin and Galgani, 2011), selective modulation of AMPK activity in the VMH could represent a strategy to target metabolic disorders characterized by alterations in energy balance such as obesity.

## Future Challenges

Hypothalamic AMPK plays a critical role in energy homeostasis control through the regulation of appetite, thermogenesis, and glucose metabolism. The hypothalamus contains different nuclei and multiple populations of neurons with specific and sometimes opposed functions. However, most of the studies implicating hypothalamic AMPK in metabolic control have not considered the diverse nature of the hypothalamus and therefore caution should be taken when interpreting the current data. In this regard, the use of targeted approaches to investigate the role of AMPK signaling cascade in specific subsets of neurons or other central nervous system cell types will help to further define its role. Another aspect to take into account is the AMPK tissue-specific differences in terms of modulatory activity and metabolic effects. This is an important issue that should be considered when designing therapeutical strategies to target AMPK.

## Conflict of Interest Statement

The authors declare that the research was conducted in the absence of any commercial or financial relationships that could be construed as a potential conflict of interest.
